# Staff experiences with implementing a case conferencing care model in nursing homes: a focus group study

**DOI:** 10.1186/s12913-019-4034-0

**Published:** 2019-03-27

**Authors:** Sigrid Nakrem, Geir-Tore Stensvik, Rickard Johan Skjong, Joan Ostaszkiewicz

**Affiliations:** 1 Department of Public Health and Nursing, Norwegian University of Science and Technology (NTNU), NO-7491 Trondheim, Norway; 2 Work Environment Unit in Trondheim kommune, Postboks 2300 Torgarden, 7004 Trondheim, Norway; 3 Centre for Quality and Patient Safety Research- Barwon Health Partnership, School of Nursing and Midwifery, Deakin University, Burwood, Victoria 3125 Australia

**Keywords:** Case conferencing, Dementia, Focus group, Geriatric assessment, Healthcare services, Implementation, Neuropsychiatric symptoms, Nursing homes, Organisation, Qualitative methods

## Abstract

**Background:**

A majority of nursing home residents have dementia, and many develop neuropsychiatric symptoms. These symptoms are often caused by neuropathological changes in the brain, but modifiable factors related to quality of care also have an impact. A team-based approach to care that include comprehensive geriatric assessments to facilitate clinical decision-making and structured case conference meetings could improve quality of care and quality of life for the residents. Despite recommendations to adopt this approach, dementia care does not reach standards of evidence-based practice. Better implementation strategies are needed to improve care. A cluster randomised controlled trial with a 12-month intervention was conducted, and the experiences of staff from the intervention nursing homes were explored in a qualitative study after the trial was completed. The aim of the present study was to describe: (i) staff’s experiences with the intervention consisting of comprehensive geriatric assessments of nursing home residents and case conferencing, and (ii) enablers and barriers to implementing and sustaining the intervention.

**Methods:**

Four focus groups with a total of 19 healthcare staff were interviewed, representing four out of eight intervention nursing homes. Thematic content analysis was used to interpret the transcribed data.

**Results:**

Two major themes emerged: 1) learning experiences and 2) enablers and barriers to implementation. The participants had experienced learning both on an organisational level: improvements in care and an organisation that could adjust and facilitate change; and on an individual level: becoming more conscious of residents’ needs and acquiring skills in resident assessments. Participants described important enabling factors such as managerial support, drivers for change, and feasibility of the intervention for the local nursing home. Barriers to implementing and sustaining the intervention were time constraints, lack of staff training, unsuitable electronic patient record system for care planning and high complexities of care and instabilities that are present in nursing homes.

**Conclusions:**

Quality improvements in nursing homes are difficult to sustain. In order to offer residents high quality of care that meet their individual needs, it is important for management and nursing home staff to be aware of and understand factors that enable or constrain change.

## Background

The majority of nursing home residents are frail older adults with complex needs due to several concurrent chronic conditions and are therefore dependent on advanced nursing care [1, 2]. The diversity of residents’ needs, which range from social care needs to palliative care needs, adds to the complexity of nursing care [3]. Many nursing home residents have cognitive impairment, which can affect their quality of life, particularly when they develop neuropsychiatric symptoms (NPS) such as aggression, agitation and depression [4, 5]. The present study explores staff experiences with an intervention that aimed to implement structured assessments of NPS and formalize care-planning meetings (case conferences) in nursing homes. A cluster randomised controlled trial with a 12-month intervention consisting of comprehensive geriatric assessments of nursing home residents and case conferencing was conducted in 17 nursing homes, and the experiences of staff from the intervention nursing homes were explored in a qualitative study after the trial was completed.

### Quality of care, quality of life and challenges in care planning for residents with neuropsychiatric symptoms

Quality of care and quality of life for long-term residents are two closely linked domains that need to be addressed in nursing homes. Quality of care in nursing homes is a multidimensional concept [3, 6], including both the technical aspects of care, leading to improved health outcomes for the residents [7], and the interpersonal interaction between residents and nurses [8]. Interpersonal skills are part of the nurse’s professional competence, and are regarded as fundamental for providing person-centred care [9]. Care that is person-centred can improve the resident’s quality of life, and involves knowing the resident and adequately addressing the person’s individual needs [10–12]. Since more than 80% of residents living in Norwegian nursing homes have a diagnosis of moderate to severe dementia [13], and as many as 90% of people living with dementia demonstrate at least one NPS during the course of their disease [14, 15], quality of care and quality of life for these residents represent major challenges to the staff. The aetiology of NPS is mostly unknown, but factors such as neuropathological changes in the brain, unmet psychosocial needs and physical health problems are thought to have an impact [16, 17]. Pain, infections, dehydration, constipation and incontinence are common health problems associated with NPS. Some of these factors relate to the quality of care and are therefore modifiable [18, 19]. Non-pharmacological interventions, i.e. psychosocial interventions should be used as the first-line treatment for the management of NPS in people with a diagnosis of dementia [5, 20]. In addition, person-centred care and individualised interventions aimed at modifying NPS and thereby maintaining quality of life are recommended [12, 16, 21]. All possible causes of NPS should be assessed in order to implement person-centred and individualised interventions that improves the resident’s quality of life [22, 23].

### Comprehensive geriatric assessment and case conferencing

The term ‘geriatric assessment’ commonly refers to evaluation of an older person performed by an individual clinician (usually a primary care clinician or a geriatrician), but it also refers to a more intensive multidisciplinary program, known as a Comprehensive Geriatric Assessment (CGA) [24]. Comprehensive Geriatric Assessment has become the internationally established method to assess older persons in clinical practice [25]. Comprehensive Geriatric Assessment is performed at varying levels of intensity in different settings, and its content may vary with the healthcare setting [26]. It involves a systematic multi-disciplinary team evaluation of the older person that identifies a variety of health problems that can be treated, and offer better health outcomes [24, 25]. The assessment is followed by the development of an individual care plan that explicitly states the older person’s goals of care, who is responsible for achieving them and a timeline for review of progress. However, the ability of CGA to improve outcomes (such as decreased hospitalization, better quality of care and lower mortality) depends on specific CGA models and the settings where they have been implemented [24]. In the present study, we used a team management approach where trained registered nurses (RNs) performed CGA to facilitate clinical decision-making before creating an individualised care plan in a structured case conference meeting in the nursing home.

Case conferencing has been used across different settings in health care [27]. It is an intervention for evaluating individual needs of residents with dementia in nursing homes, and it has positive effects on NPS [28]. A qualitative study on case conferences showed that case conferences could also facilitate communication and coordination between the staff [29]. Regular case conference meetings provide opportunities for nurses to practice reflective communication in a structured, goal-oriented way, creates a common understanding of the case, and thereby nurses can identify the individual needs of residents with dementia and agree on individualised care interventions [30]. Despite these recommendations, reviews show that the dementia care globally does not reach standards of evidence-based practice [31]. Implementation strategies are therefore necessary to enhance the uptake of the positive effects of CGA and case conferences.

### Implementing and sustaining changes in nursing homes

Implementing change in nursing home practice is challenging, and it has been demonstrated that the provision of care based on best evidence in the care of older adults, especially in nursing home settings, is low [32, 33]. This might be explained by a lack of understanding about the complexity of nursing home care, and lack of awareness of the link between the individual nurse’s practice and organisational factors [34–36]. Improvements in care practices based on learning involves adjusting individuals’ attitudes and behaviours, however, for learning to become organisational, new insights must become distributed among the organisation’s members as shared understandings or shared mental images [37]. Implementing and sustaining change means that the new practice is integrated into routine nursing home care [38]. A range of implementation strategies are needed to succeed. It involves, among others, spread of research and guidelines on for instance CGA to nursing homes in general, and active, targeted interventions in each nursing home [38]. Implementation science focuses on challenges associated with the uptake of evidence into practice to improve quality of care [39]. There is a considerable body of knowledge on how to change health care personnel’s behaviours in general [40–42], but implementation research in nursing homes is still lacking. Various implementation activities are recommended [43], but active approaches and multi-dimensional interventions are more effective than passive approaches and single interventions [33, 44]. In addition, implementing new practices should be guided by evaluating potential enablers and barriers for the change when planning the implementation strategy [40, 44].

### Aim of the study

The aim of the study was to describe nursing home staff’s experiences with an intervention consisting of comprehensive geriatric assessment and care planning in structured case conference meetings. Further, the aim was to obtain a deeper understanding of the enablers and barriers to implementing and sustaining the intervention.

## Methods

### Setting and participants

The present study is part of a larger study on quality improvements in nursing homes (see for example publications [45–49]) connected to the university’s priority research area ‘Ageing and older people’s health’ (https://www.ntnu.no/ism/aldring#/view/about). One part of the larger study was a c-RCT, testing an intervention involving CGA and regularly case conferencing based on these assessments and group discussions (for details, see registration at ClinicalTrial.gov - NCT 02790372, results will be reported elsewhere). Eight nursing homes in Mid-Norway, including 159 residents (all > 65 years), were randomly selected to the intervention. The nursing home management and RNs in the intervention nursing homes received training to perform assessments and the case conferences (see Table 1 for details), and then included all nursing staff in their units. Nine control nursing homes (150 residents) performed care as usual, and are not included in the present study. Focus group methodology is well suited to discover what influences behaviour and satisfaction with a service [50]. Nursing staff at the eight nursing homes that performed the intervention were invited to participate in focus group interviews to share their experiences of participating in the intervention. Only nursing staff working in the clinical setting were invited to participate in the interview. The reason for not including management was to avoid power inequalities in the groups and to enable the nursing staff to voice their opinion on nursing home leadership. Staff from four nursing homes were willing to participate.

**Table 1:** Overview of the intervention

Element of intervention	Content	Training and support
Comprehensive Geriatric Assessment (CGA)	Neuropsychiatric inventory-Questionnaire (NPI-Q) Cornell scale for depression in dementia (CSD) The quality of life in late-stage dementia (QUALID) Clinical Dementia Rating (CDR) scale The physical self-maintenance scale (PSMS)	A 30 min’ lecture on how to use assessments for case conferencing. Written educational material.
Case conferences	Four structured steps: 1) Evaluate effects of previous nursing interventions based on updated patient assessment 2) Create a common understanding of the problem or area for improvement 3) Determination of concrete and realistic goal of care (SMART)^a^ 4) Discuss, decide and define nursing interventions and appropriate method for evaluation	A 45 min’ lecture on symptoms, causes and explanations of neuropsychiatric symptoms A 45 min’ lecture on why and how to perform a case conference. A 30 min’ practical training session in performing a case conference (using a resident case from the actual nursing home as example). Written educational material and a manual for structuring the case conference.
Documentation and reporting (using Electronic Patient Record)	Care plan should be updated after each case conference by updating the electronic patient record (nursing module)	A 45 min’ lecture on the nursing care process including demonstration of resident example
Additional assessments (when the resident’s symptoms/needs, or situation requires it)	The brief agitation rating scale (BARS) 24-h registration of behaviour form	A 30 min’ lecture on how to use assessments for case conferencing. Written educational material.

^a^SMART: the goal should be Specific, Measurable, Assignable, Realistic and Time-related

### Data collection

To collect data, four focus groups were organised, one from each of the nursing homes that consented to participate. The focus groups consisted of 3–6 participants each, including 6 RNs (some with advanced education in geriatrics), 12 Licenced Practical Nurses (LPNs) and 1 Nursing Assistant (NA), in total 19 participants. All participants had experienced the intervention for at least 12 months and had practiced case conferences as organisers, chair of the conferences and/or active members of the clinical care team. Three of the participating nursing homes had successfully performed the intervention as described in the c-RCT protocol, but one nursing home had not had regular case conference meetings often enough to be considered to be following the intervention per protocol. The focus group interviews took place in March and April 2017. All interviews were performed in a meeting room in each of the participating nursing homes. The interviews lasted for approximately 60 min. All interviews were held during participants’ working hours, and their ordinary tasks during the time spent were taken care of by other healthcare staff. A semi-structured topic guide with key questions was used during the interviews (see Table 2). The topic guide was developed by the research team based on theory, previous research and clinical experiences with implementation processes in healthcare services. The interviews were moderated by one of researcher who led the interview (SN in three of the focus groups, GTS in one), and another researcher participated as observer, took notes of the groups’ communication style and asked additional questions when needed (GTS in three of the focus groups, RJS in one). The focus group discussions were audio recorded to ease transcription and analysis of data; in three cases by using video camera and in the last only audio recorded because the participants did not consent to video filming.

**Table 2:** Topic guide

Topic	Key questions
Introduction	Can you describe how you started with the intervention?
Your experiences of being involved in the intervention	How did you plan the case conferences (including patient assessments)? How did you experience performing meetings? What roles did the different meeting participants perform? How did you reach consensus?
Positive and negative aspects of the interventions	How did you experience patient assessment procedures? How did you experience individualised care planning? What barriers to implementation did you experience? What did you learn by participating in the intervention?
Factors we should consider if we were going to implement this on a broader scale	Have you used assessment and case conferences for the residents after the intervention period? Why/why not? Can you mention one or two key factors important for success?
Closure	Do you have anything to add that has not been mentioned? How did you experience participating in this focus group?

### Analysis

All interviews were transcribed verbatim, leaving out personal information in order to preserve the participants’ anonymity. Thematic content analysis as described by Graneheim and Lundman (2004) [51] was used to analyse data. First, the text was read through several times to get a sense of the whole. Next, meaning units were identified, and sorted into one of the two main objectives or themes. The meaning units were then condensed into a description close to the text, retaining the manifest content, or, when applicable, condensed into an interpretation of the underlying and latent meaning. The condensed text was coded and abstracted into sub-categories. Lastly, the sub-categories were unified into the main categories within the two main themes. All authors participated fully or partially in the analysis process, and we met to discuss the interpretations. Any disagreements were thoroughly deliberated and consensus about the final sub-categories and categories was reached [51].

### Ethical considerations

The Norwegian Centre for Research Data (NSD) approved the project (project no. 51795, 2017). All participants were handed out written information about the study and all gave their written consent to participation. The participants were informed that they could withdraw from the study at any time, and that their information then would be omitted in the analyses. Before publishing the findings, data were anonymised, and quotes used in this article are presented without personal information to avoid potential for recognising individual participants.

## Results

The findings are sorted into two themes: 1) *Learning experiences*; and 2) *Enablers and barriers to implementation*. Within each theme, categories and subcategories are presented below.

### Learning experiences

Participants spoke freely about both negative and positive experiences. Learning experiences were divided between learning on the organisational and the individual level. Table 3 shows an overview of the sub-categories and codes related to what the interviewees stated they had learned during the intervention period.

**Table 3:** Learning experiences sub-categories and related codes

Category	Sub-category	Codes	Illustrative quotes
Organisational learning	Quality improvement of care	Care improvements Effect of care Consensus	*In reality, we observed a positive effect on patients, I would say. Everyone worked together towards a common goal.* *When something is decided, then we should pull in the same direction, it is important that everyone join in on the decided actions.*
	Facilitation	Assure structure Shared experiences Idea development Learning together	*When we had the structured meetings, I did not feel that …* [the meeting slipped off course] *in this meeting, we kept the structure.* *You have to think in new ways. Maybe we’re not very good at that, that more of us sit together and think out loud.* *The point was not just to intervene on the included patients; we were also supposed to learn to have structured meetings, weren’t we?* *These kinds of meetings are useful,* […], *we may have different information about the patient and see things differently, at the same time we are supposed to agree on one thing that could benefit that patient, and systematize our efforts.*
	Adjustments	Local practice Prioritisation of time used	*I wonder if we have worked differently, some have done one measure, but we in* [unit] *C took the whole patient and went through all the measures during the meeting. Obviously, this lasted more than 20 min.* *Initially we met towards the end* [of the shift], *then we realized that maybe 12 o’clock was the best time … so that everyone did not withdraw* [from the ward] *at the same time.* *We become so consumed by our daily routines, so the best time to meet is actually after two o’clock.*
Individual learning	Personal development	Consciousness Reflection Motivation Engagement	*Actually, we have always formulated care plans and such, but when we had those meetings, we explored the patient’s situation more deeply. You become more thoughtful on the things you are doing.* *I observed progress, very satisfying to see that it was possible.* *When you’ve had sufficient training, like us, then you may also feel inspired.* *You become more compassionate when you try to withdraw a bit and observe it from a bird’s-eye view, and look into causes of why things turn out this way or that way.*
	Improved skills	Resident assessments Documentation of care	*What I see mostly, is that many unjustified opinions have stopped, at least in the care plans. What is documented, has become much more concrete.* *Likewise, with the update of information on the computer, it was very helpful to do that simultaneously during the meetings.* *We wrote down the care that was decided. This was pretty detailed and concrete, and then everyone had to do it in the same way.*

On the organisational level, all participants believed that the care quality had improved because of regularly performing assessments and case conferences. Effective interventions and timely evaluation of nursing interventions was the main reason why they thought that the residents’ needs or problems were better handled after being part of the trial. A main feature of the intervention was that the nursing staff had to agree upon a care plan and document it in the electronic patient record (EPR) before ending the meeting. Thereby, all staff, not only those who had participated in the meeting, could be updated on changes in the individual care plan, and all staff performed the care in a consistent manner.

Another reported benefit of the trial was that it facilitated case conferencing. The case conferences were regarded as an efficient way to share care experiences in a structured manner. Participants felt that ideas on nursing interventions could develop, they reflected upon the individual resident needs and made decisions that felt best suited the resident. The participants’ opinion was that the case conferences facilitated shared learning experiences that stimulated and improved professional practice.

However, participants from all four nursing homes emphasised the need to adjust some aspects of the intervention. These were related to the necessity to align the case conferences to existing ward meeting schedules. Some wards made changes in the way they prioritised the time used for case conferences and other care planning activities.

On an individual level, participants indicated that participation in the trial had made them a better caregiver and had influenced their personal development. Participating in the trial was perceived as engaging and made participants more conscious about their tacit care practices. The intervention, especially the resident assessments, had supported a more person-centred perception of the resident, and the staff became more reflective upon their attitude towards the residents in general. Performing better care motivated participants to continue to develop their competence in care.

In addition, participants indicated that participating in the trial had enhanced their nursing skills related to needs assessment and the importance of care planning and documentation of care plans. Many of the interviewees in the focus groups stated that such comprehensive assessments of their nursing home residents had not been done previously on a regular basis. Sometimes it was requested from the nursing home physician. The nurses felt more competent to perform assessments and interpret the data. Even though the assessments were time-consuming, especially in the beginning, the time spent was felt to be very helpful for the case conferencing and to develop an individual care plan. The different nursing homes had software for care plans in the EPR installed; however, the EPR was used to a varying degree to update resident’s needs and to create an individual care plan. Being triggered to update the care plan during the case conference meeting also motivated staff to learn to use the EPR properly, and it increased trust that the care plan in the EPR was updated.

### Enablers and barriers to implementation

**Fig. 1:**
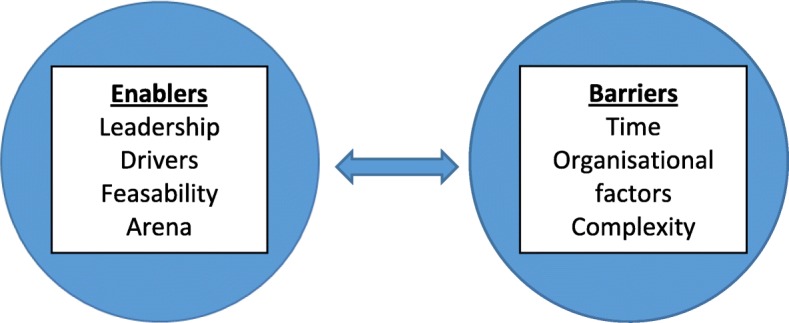
Enablers and barriers to implementation

One of the main objectives in the study was to engage with participants to seek advice about how to implement the CGA and case conference intervention. The sub-categories are shown in Fig. 1.

#### Enablers to implementation

Participants in the focus groups described varying levels of managerial support for the intervention. Specifically, two of the nursing homes had an engaged management that participated and supported the nurses during the trial. In the two others, the participants felt that the nursing home director had imposed the c-RCT upon them, and then left them to find out themselves how to manage it. Participants from one nursing home expressed:

*I think it is a joint responsibility; a leader responsibility to settle some guidelines for who is responsible for the quality of care, and everyone’s responsibility to demand qualifications of the staff.*

In contrast, a participant from another nursing home spontaneously expressed: *No, I was thrown right into it.* Both strategies had worked, but those who had performed the c-RCT independently wanted more support from the managers, for instance an understanding that more competent nursing staff was required. Another perceived enabler for implementation was sufficient skills for planning and chairing a case conference meeting:

*I think it has to do with who’s chairing the meeting, if you don’t say “welcome, now we have called for a meeting” … then people start chatting about everything else.*

Moreover, the training and educational material provided by the researchers at the beginning of the trial was considered as very helpful: *If everyone comes well prepared to the meeting, it is easier for everyone*. Practicing case conferencing regularly had also helped them to implement the meetings, and they emphasised that learning this necessarily took time.

Secondly, the participants discussed factors that could optimise changes in practice related to care for residents with dementia and NPS. Participating in a research trial had enhanced the nursing staff’s commitment to do what was expected of them from the researchers. A few of the participants felt that participation in the c-RCT was decided from the nursing home manager, and that they therefore were obliged to perform the trial. However, as the nursing staff experienced CGA and case conferencing as a useful way of structuring care planning and the new model could improve the residents’ situation, the nursing staff soon experienced a sense of ownership of the trial that increased their motivation:

*I think that a lot is solved if all staff get enough information from the start. If it is emphasized that now we are doing things this way, perhaps people get the feeling that you must, should …*

During the case conference, the staff were expected to reach consensus about care plans, and this was perceived as a key factor for staff’s commitment to what was decided in the meetings. They saw that the case conferences had certain weight when it came to determine how to perform care in a consistent and professional manner, and all staff trusted the change to be better for the residents: *Yes, it felt as if everyone was more committed to documentation and follow up on the care plan, whether it worked well, or not.* As well, external sources, such as negative press from local newspapers, or complaints about care from the public or family members, were drivers for change. Such external drivers influenced both nursing staff and managers, as one participant expressed:

*It was the management at our nursing home who initiated it* [improvements in residents’ nutrition]*. Because there were employees from one ward who went to the media and told that they did not have time to feed the residents. It became an issue in the local paper, which was somewhat unfortunate. That’s when it started, but it’s actually a good thing.*

The third category of enablers was related to care plans, documentation and evaluation of care for the individual resident. Doing CGA was a prerequisite for determining the resident’s needs and what problem to discuss in the case conference. This was also the basis for decision on a care plan that was suitable for the resident and that could meet that particular resident’s needs, as one participant expressed: *The important thing is to keep the resident in focus and accomplish those measures where it is achievable.* Another participant expressed: *The assessments must be less complicated, it needs to be simplified, and you should explain that the assessment instruments are used to improve care for the residents*. Having EPR software that supported documentation of detailed care plans and that had definite methods for evaluating the care on measurable endpoints was essential: *Yes, we will get nowhere with starting up care interventions if they aren’t documented in the EPR.*

Finally, the participants were very satisfied with the case conference meetings because the meetings created an arena to discuss residents’ problems and needs professionally. The time and space the trial gave the nurses to perform assessments and discuss the residents’ problems was perceived as very useful, expressed by one participant:

*The thing that delivered the project was to buy time. When we had meetings, those on the evening shift came an hour early, two o’clock instead of three o’clock, so nothing interfered with ordinary tasks.*

The meetings helped the nurses to structure and order their busy days in the ward. Those who had been able to set up meetings regularly were most successful, indicating an organisational change or learning. Spending sufficient time in the beginning to learn to do CGA and case conferences was considered by the participant a priority: *When you have a new system, it is not done in a fortnight. Time is what it’s about, I think. I think one should consider that things take time.* As well, the participants perceived that the case conferencing method facilitated openness to discuss the residents’ problems, discuss their care and accept disagreements.

#### Barriers to implementation

Several barriers to implementation of the change in practice were identified from focus group discussions. Time constraints was the most common barrier. The nursing staff stated that, at times, it was too busy in the ward to sit down and discuss the care problems. Especially when they had new residents or residents’ care needs increased, the staffing level did not allow time for meetings. At the same time, the participants agreed that it might be a question about prioritisation between tasks during a day, for instance if a nurse was cleaning cupboards instead of doing CGA:

*We generally have poor nurse coverage here and the flat hierarchy where everyone is supposed to do everything, like washing cupboards and windows, means that we are not able to do everything. So, this professional way of actually doing things is both in demand and overlooked.*

Many of the barriers were related to organisational factors. The intervention was perceived to be relatively complex, and many nurses felt they were not competent to perform CGA. The availability of staff with the skills to chair the meetings was another barrier. For instance, working part-time inhibited continuity, and staff turnover or sick-leave/maternity leave made it necessary to repeat the training. Lack of stability influenced staff’s working conditions, as new staff needed to be trained and those who had worked in the same ward felt extra strain from the continual change in staff: *We were quite good in the beginning, but then it declined. It did not take much in terms of work on the wards or shortage of nurses before it became difficult to prioritise*. In addition, a lack of adequate functioning EPR and sufficient number of computers hindered documentation of care plans, which was a prerequisite for the intervention.

The participants expressed that the implementation of the intervention was hampered due to complexity of their work environment. Many of the residents stayed for a short time, and their health conditions could change rapidly.

[We had] *many very sick residents, then came the summer vacation time, and it just dwindled away I think. This is something that bothers me … perhaps one’s bad conscience that things weren’t done well enough, and that I should have been a stronger driving force for more meetings … and such.*

The participants described a lack of control over the working environment, the complexity of residents’ care needs, the composition of residents and instability of nursing staff, and how this could be a barrier for maintaining an adequate intervention over time. They were not prepared for the time it took to develop skills to perform CGA and case conferences, and change was not visible for some time. The staff’s conflicting perspectives on what nursing home residents’ care needs are, challenged the multidisciplinary cooperation, for instance with the physician or the physiotherapists. Some participants indicated that it was difficult to remain patient and motivated, and support from management and all staff was essential: *I felt that the feedback was that this was something the nurses should handle. This affected the motivation of the others.* Other participants thought that one reason for not succeeding with the implementation could be fear of change or lack of openness to innovations in the organisation or individuals: *It’s a bit about resistance to the EPR, and people who resist writing*.

To sum up, the present study describes the participants’ learning experiences on both organisational and individual levels. According to the participants, implementing CGA and case conferences improved quality of care and facilitated organisational learning. On the individual level, the participants described experiences of personal development and improved skills. Important enablers for the implementation of a care model including CGA and case conferences were the support from management, the presence of drivers such as commitment, the feasibility of the new care model and that the nursing home develops as an arena for change. Barriers for implementation were related to time constraints, and organisational factors in the nursing home such as lack of staff training and suitable EPR. The high complexity of care and instabilities that are present in nursing homes hindered continuous implementation.

## Discussion

The nursing home service is resource demanding when it comes to competence and time [52]. Therefore, it is important to have good structure to ensure that the resources are used efficiently and at the same time, is of high quality. Although the majority of nursing home residents are frail, and have complex health conditions, including advanced dementia, with short longevity [13], the participants in the study experienced that the intervention had an impact on quality of care. Better resident assessments and more structured meetings were features of the intervention that were perceived as essential factors for the improvements. In addition to advanced nursing care, nursing home residents require care that is person-centred, including a comprehensive care plan [53]. The participants perceived the CGA and case conferences were a means to know the residents better and more precisely address their individual needs. Case conferencing may contribute to greater involvement of health personnel in nursing planning [54]. When facing challenges in care related to NPS in residents, the motivation for staff to learn how to improve care is probably high. Learning to use assessment instruments targeted to the individual resident’s problems, and having structured meetings to discuss the cases can address these challenges and improve quality of care [29].

Implementation strategies most often aim at changing individual staff member’s knowledge, attitudes or behaviours [44]. However, organisational change and learning are equally important, since quality of care is not influenced by the performance of individual healthcare worker alone [55]. Case conferencing might facilitate both organisational learning and personal development, by offering a mechanism for participants to share their knowledge in meetings, learn to use resident assessments and agree on a care plan. The intervention in the c-RCT was advanced and complex, and was originally targeted to the RNs. However, because of the high turnover of RNs and managers it was necessary to adjust the intervention to additionally target LPNs and NAs. Each nursing home is different in the way it is organised and the physical environment, therefore a key learning from the research was to support the local nurses to modify the intervention, even if some parts of the intervention requires higher competence [56, 57]. Moreover, minimising a top-down and externally led approach allows staff to adjust the components of the intervention to their local context [37, 58–60].

The present study showed that good information at the start of the trial and an understanding of the importance of performing the trial as planned enhanced commitment from the staff. However, the participants expressed that support from the leadership was also essential for success; for instance understanding from the managers on how to activate and use the nursing competence adequately to improve quality of care. In addition, the participants emphasized that all staff had to understand that the nurses needed time to use their competence. In nursing homes, barriers to implementation of care improvements include a range of factors such as lack of knowledge, staffs’ attitudes and beliefs, understaffing and weak management [33]. To overcome such barriers, the whole organisation from institution owner (the municipalities) to the individual healthcare worker needs to be involved. Organisational change is a continuous learning process, not a one-time event [37, 40]. If all staff members and the management identify the organisation’s own problems, understand the reasons for change and feel involved in decision-making, it is more likely that the nursing home will function with shared values and improve residents’ outcomes [58, 61–63].

There are some limitations of the study that needs to be considered. As the focus groups were limited to staff from four nursing homes in Norway, the findings are not generalizable to all nursing homes. At the same time, the randomisation process ensured a representative sample of Norwegian nursing homes. Participants from the management were not included in the study, and exploring the managers’ perspectives is warranted in further work. Transferability of the findings was strengthened by the description of the setting and including quotes from the participants, thus, enhancing the reader’s possibility to determine to which extent the findings can be applied to another situation [64]. Another consideration is the threat to the trustworthiness of the findings posed by the researchers’ presuppositions that could have affected the interpretation of the qualitative data [64]. Since one of the researchers who conducted the focus group interviews was also responsible for training for the c-RCT and data collection, he had to reconcile both qualitative and quantitative epistemological stances. This threat was countered by his reflexivity and the involvement of two or more researchers from varying backgrounds to individually and collectively discuss the emerging themes and codes.

## Conclusion

Changes in organisations are difficult to sustain, even if the staff experience quality improvements in trials. Barriers such as lack of continuity in staff and the complexity in the service are not easy to overcome. Enabling resources such as commitment and culture for change might be present in the nursing home, but they need to be cultivated to flourish. The responsibility to improve care practice rests with everyone engaged in the services for nursing home residents, from the policy level, the management and the individual care worker. In the years to come there will be an increasing demand for high quality nursing home services. Society as a whole must recognize the need for action to enhance residents’ experiences of nursing home services, to use limited resources wisely and to offer services that are person-centered to those who need it. This calls for quality management and continuing education of nursing home staff that enable them to engage in quality improvements that meet nursing home residents’ individual needs.

## Abbreviations

BARS: Brief Agitation Rating Scale
CDR: Clinical Dementia Rating scale
CGA: Comprehensive Geriatric Assessment
C-RTC: Cluster Randomised Controlled Trial
CSD: Cornell scale for depression in dementia
EPR: Electronic Patient Record
LPN: Licensed Practical Nurse
NA: Nursing Assistant
NPI-Q: Neuropsychiatric Inventory Questionnaire
NPS: Neuropsychiatric symptoms
NSD: Norwegian Centre for Research Data
PSMS: The physical self-maintenance scale
QUALID: The quality of life in late-stage dementia
RN: Registered Nurse
SMART: Specific, Measurable, Assignable, Realistic and Time-related
